# Attenuating Radiation Exposure, Understanding the Mechanisms of Radiation-Induced Toxicity, and the Development of Radiation Countermeasures

**DOI:** 10.3390/toxics11040306

**Published:** 2023-03-25

**Authors:** Wanchang Cui, Mang Xiao

**Affiliations:** 1 Armed Forces Radiobiology Research Institute, Uniformed Services University of the Health Sciences, Bethesda, MD 20889, USA; 2 The Henry M. Jackson Foundation for the Advancement of Military Medicine, Inc., Bethesda, MD 20817, USA; 3 Department of Pharmacology and Molecular Therapeutics, Uniformed Services University of the Health Sciences, Bethesda, MD 20814, USA

Radiation exposure is a complex issue that has both benefits and risks for human health. While it is a vital component of many medical imaging technologies and cancer treatments, it can have side effects for patients. Furthermore, radiation is a serious threat to public health in the event of industrial accidents or nuclear device detonation. To address these concerns, the latest research on radiation exposure and biological effects has been compiled into a Special Issue of *Toxics*.

This Special Issue, featuring eight research and two review articles, covers a wide range of topics related to the effects of radiation exposure on human health. Leading authors across the world from different universities/institutes have contributed their time and expertise in radiation-related biology, physics, physiology, and pathology to provide new information on this topic. The articles can be roughly divided into two sections: (1) physical approaches to quantify and attenuate radiation exposure and (2) biological studies investigating radiation toxicity and radiation countermeasure development.

In the first section, Suliman et al. revealed the need for vital radiation protection considerations due to neutron contamination in external beam therapy [1]. Masoud et al. presented different serpentine rocks that can be alternatives to radiation-shielding concrete [2]. Smith et al. provided valuable data to set the occupational exposure limits for activities involving tritiated steel particles [3].

In the second section, Cui et al. studied pharmacokinetics and its effects on cytokines of a potential radiation mitigator, IL-18 binding protein [4]. Lenarczyk et al. showed the importance of T cells in non-targeted heart disease following irradiation [5]. Wang et al. demonstrated the contribution of radiation-induced inflammatory responses to skin wound healing [6]. Simmons et al. provided insights into the effects of fractionated proton irradiation on spatial and short-term memory [7]. Zhang et al. reported the safety of soluble melanin and melanin-producing bacteria [8]. Sharma et al. reviewed the current understanding of endothelial dysfunction following radiation exposure [9] and Russ et al. reviewed the differences in medical uses and cellular effects between high and low linear energy transfer radiation [10].

Overall, this Special Issue provides readers with an understanding of the latest advancements in radiation attenuation, mechanisms of radiation toxicity, and the need for ongoing research on mitigation strategies to address the potential health effects of radiation exposure on both patients and the general public. The compilation of research and review articles from leading experts in the field makes it an essential read for anyone interested in the impact of radiation exposure on human health.
